# Insights into the Molecular Mechanisms Underlying Mammalian P2X7 Receptor Functions and Contributions in Diseases, Revealed by Structural Modeling and Single Nucleotide Polymorphisms

**DOI:** 10.3389/fphar.2013.00055

**Published:** 2013-05-07

**Authors:** Lin-Hua Jiang, Jocelyn M. Baldwin, Sebastien Roger, Stephen A. Baldwin

**Affiliations:** ^1^School of Biomedical Sciences, Faculty of Biological Sciences, University of LeedsLeeds, UK; ^2^INSERM U1069 Nutrition, Growth and Cancer, Université François-Rabelais de ToursTours, France

**Keywords:** extracellular ATP, P2X7R, structural modeling, ATP-binding, ion channel, large pore, NS-SNPs

## Abstract

The mammalian P2X7 receptors (P2X7Rs), a member of the ionotropic P2X receptor family with distinctive functional properties, play an important part in mediating extracellular ATP signaling in health and disease. A clear delineation of the molecular mechanisms underlying the key receptor properties, such as ATP-binding, ion permeation, and large pore formation of the mammalian P2X7Rs, is still lacking, but such knowledge is crucial for a better understanding of their physiological functions and contributions in diseases and for development of therapeutics. The recent breakthroughs in determining the atomic structures of the zebrafish P2X4.1R in the closed and ATP-bound open states have provided the long-awaited structural information. The human *P2RX7* gene is abundant with non-synonymous single nucleotide polymorphisms (NS-SNPs), which generate a repertoire of human P2X7Rs with point mutations. Characterizations of the NS-SNPs identified in patients of various disease conditions and the resulting mutations have informed previously unknown molecular mechanisms determining the mammalian P2X7R functions and diseases. In this review, we will discuss the new insights into such mechanisms provided by structural modeling and recent functional and genetic linkage studies of NS-SNPs.

## Introduction

It has been well established that extracellular ATP is a widely used signaling molecule in both excitable and non-excitable cells (Burnstock et al., 2010; Burnstock, 2012). As one key constituent mediating extracellular ATP signaling, mammalian cells express a family of P2X proteins, P2X1–P2X7, which form homo/hetero-trimeric ionotropic receptors at the cell surface exclusively responding to extracellular ATP (North, 2002; Khakh and North, 2006). Many studies over the past decade, and in particular those using transgenic mice, have gathered a large volume of evidence to show pivotal roles for the mammalian P2XRs in a wide range of physiological and pathological processes (Surprenant and North, 2009; Jiang, 2012; Kaczmarek-Hajek et al., 2012). The mammalian P2XRs have been also subject to extensive site-directed mutagenesis studies, which, with the aid of the recently determined atomic structures of the zebrafish P2X4.1R in the closed and ATP-bound open states (Kawate et al., 2009; Hattori and Gouaux, 2012), have significantly enriched our understanding of the molecular mechanisms that determine the common functional properties of the mammalian P2XRs: ATP-binding, ion permeation, and channel gating (Browne et al., 2010; Evans, 2010; Young, 2010; Khakh and North, 2012; Jiang et al., 2013).

The P2X7 receptor (P2X7R) is unique among the P2XR family, exhibiting distinctive or hallmark pharmacological and functional properties (Jiang, 2009). The concentration of ATP required to activate the P2X7R is one or two orders of magnitude greater than for the other P2XRs. 2′-(3′)-*O*-(4-benzoylbenzoyl)-ATP (BzATP), a synthetic ATP analog, is more potent at the P2X7R than ATP, whereas the reverse order of potency is true at the other P2XRs (North and Surprenant, 2000). The P2X7R confers cells with an exceptionally dynamic permeability. Brief activation opens a canonical ligand-gated ion channel selectively permeable to small cations (Surprenant et al., 1996), and the P2X7R ion channel displays remarkable facilitation or sensitization upon repetitive agonist applications (Roger et al., 2008, 2010a; Yan et al., 2010). It is the prototypic ionotropic receptor that induces formation of a pore capable of passing much larger molecules in response to prolonged or repetitive activation; formation of such a large pore is often shown by influx and intracellular accumulation of extracellular fluorescent dyes, such as YO-PRO-1 and ethidium, or a progressive increase in membrane permeability to large organic cations, such as *N*-methyl-d-glucamine (Surprenant et al., 1996; Jiang et al., 2005). The mammalian P2X7Rs are highly expressed in immune cells and also in other cell types such as glial, neuronal, epithelial, and bone cells, where they have a crucial role in mediating extracellular ATP signaling in release of cytokines (e.g., interleukin 1β) and proteases (e.g., cathepsins), neuron-glia interactions, exocrine gland secretion, and bone remodeling (Jiang, 2012). There is also accumulating evidence to support a causative role for altered expression and function of the mammalian P2X7Rs in a number of pathologies including arthritis, chronic pain, and cancers (Donnelly-Roberts and Jarvis, 2007; Roger and Pelegrin, 2011; Adinolfi et al., 2012; Baroja-Mazo and Pelegrin, 2012; Di Virgilio, 2012; Jiang, 2012). The past few years have witnessed enormous high throughput screening efforts that have led to discovery of a number of structurally diverse, selective, and potent P2X7R antagonists; some of these molecules exhibit desirable drug-like properties and therapeutically relevant efficacy in studies using rodent disease models (Guile et al., 2009; Gum et al., 2012; Jiang, 2012). Natural compounds isolated from plants used in traditional medicines have also been shown to selectively inhibit the P2X7Rs (Liu et al., 2010; Jelassi et al., 2013). Thus, the human P2X7R has become a promising drug target. The recent clinical trials of the first two P2X7R antagonists have however been disappointing (Keystone et al., 2012; Stock et al., 2012), clearly exposing the need for a better understanding of P2X7R-mediated disease mechanisms. A clear delineation of the molecular determinants for the functional properties of the mammalian P2X7Rs, including the well-documented species differences that have serious implications for translation of preclinical animal studies into clinical testing, is hugely desirable for development of small chemical molecules targeting the human P2X7R as therapeutics. The *P2RX7* gene encoding the human P2X7R exhibits widespread single nucleotide polymorphisms (SNPs), and among them there are a large number of non-synonymous single nucleotide polymorphisms (NS-SNPs) that change the amino acid sequence and result in a repertoire of human P2X7Rs with point mutations. The gene for the mouse P2X7R (*p2rx7*) also contains NS-SNPs. Recent efforts in characterization of such naturally occurring P2X7 mutant receptors have revealed, on one hand, previously unknown molecular mechanisms for the mammalian P2X7R functions and, on the other, an association of NS-SNPs with altered susceptibility to various diseases. Studies of the NS-SNPs associated with diseases have also shed new light on the underlying disease mechanisms.

The review will provide an up-to-date overview of the structure-function relationships of the mammalian P2X7Rs using structural models based on the atomic structures of the zebrafish P2X4.1Rs, and discuss the recent progress from studies of NS-SNPs in understanding the molecular mechanisms that determine the functional properties of the mammalian P2X7Rs and their contributions in diseases.

## An Overview of the Structure-Function Relationships

### P2X7R proteins

**Figure 1:**
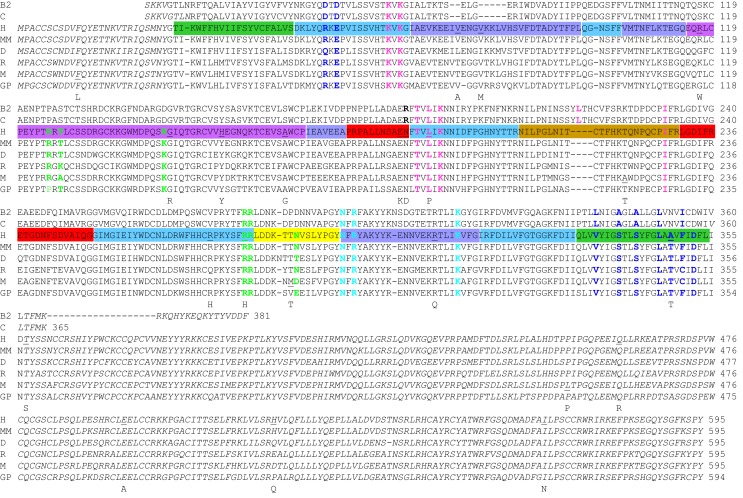
**Sequence comparison of the zebrafish P2X4.1R and mammalian P2X7Rs**. The amino acid sequences of the mammalian P2X7Rs are aligned with the sequences of the truncated zebrafish P2X4.1Rs with determined atomic structures (Hattori and Gouaux, 2012). The ΔP2X4.1-B_2_ (B2) consisting of Ser28-Phe381 with changes at three positions (C51F, N78K, and N187R) and the ΔP2X4.1-C (C) Ser28-Lys365 with changes at two positions (N78K and N187R) were used to determine the structures in the closed (PDB accession number: 4DW0) and ATP-bound open states (4DW1), respectively. The species abbreviations are: H, human; MM, macaque monkey; D, dog; R, rat; M, mouse; GP, guinea pig. The sequences in italics are absent in the atomic structures or structural models. The same color scheme is used to indicate the different domains in the human P2X7R sequence here and the dolphin-shaped single subunit structural models shown in the following figures. The nine residues in the mammalian P2X7Rs (10 in the zebrafish P2X4.1R, including Leu217) involved in forming the inter-subunit ATP-binding site (shown in Figure 3B) are highlighted with six residues from one subunit in pink and another three residues from the complementary subunit in cyan (shown in Figure 4). The residues in green are present in the structural regions surrounding the ATP-binding site (also shown in Figure 4), changes of which alter the agonist sensitivity or other functional properties of the P2X7Rs (see text for details). The residues contributing to the transmembrane ion-conducting pathway (shown in Figure 5B) and residues in the extracellular lateral fenestrations are indicated in blue. The underlined residues in the human and mouse P2X7Rs (shown in Figure 6) are mutated by NS-SNPs to the residues shown underneath.

The P2X7Rs from six mammalian species (human, rhesus macaque monkey, dog, rat, mouse, and guinea pig) and two non-mammalian species (*Xenopus* and zebrafish) have thus far been characterized in isolation to various extent (Surprenant et al., 1996; Chessell et al., 1997; Rassendren et al., 1997a; Paukert et al., 2002; Kucenas et al., 2003; Fonfria et al., 2008; Roman et al., 2009; Bradley et al., 2011b). As shown in Figure 1, all the mammalian P2X7R subunits comprise 595 amino acid residues except for the guinea pig P2X7R subunit, which is one residue shorter due to omission of Asp77 (in the human P2X7R numbering, used from this point onward). The dog P2X7R subunit contains an asparagine residue between positions 281 and 282 and lacks the residue corresponding to Thr541 in the human P2X7R subunit. The *Xenopus* and zebrafish P2X7R subunits contain 553 and 596 residues, respectively. In a pair-wise comparison, the human P2X7R subunit shares 96% sequence identity with the monkey P2X7R subunit, 78–85% with the other four mammalian P2X7R subunits, and 50–56% with the *Xenopus* and zebrafish P2X7R subunits (Bradley et al., 2011b).

All the P2XR subunits including those of P2X7Rs consist of a large, glycosylated, and cysteine-rich extracellular domain, two transmembrane domains (TM1 and TM2), a short intracellular N-terminal domain and an intracellular C-terminal domain of variable length (North, 2002; Kawate et al., 2009). The C-terminal domain of the P2X7R subunits contains between 70 and 220 more residues than that of the other P2XR subunits and thus is significantly longer. The TM1 and TM2 domains in the human P2X7R comprise residues Thr28-Ser47 and Asn332-Leu354, respectively (shown in green in Figure 1).

### The structural models of human P2X7R

**Figure 2:**
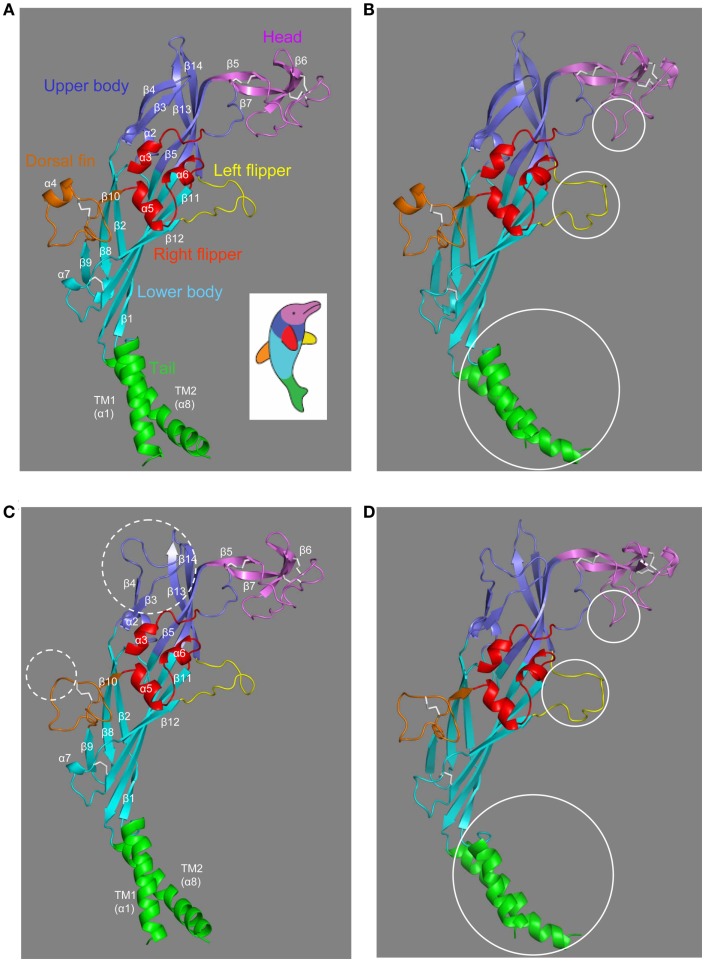
**The dolphin-like architectures of the zebrafish P2X4.1R and human P2X7R subunits**. **(A,B)** The structures of the zebrafish P2X4.1R subunit in the closed **(A)** (PDB accession number: 4DW0) and ATP-bound open states **(B)** (4DW1), respectively. **(C,D)** The structural models of the human P2X7R subunit in the closed **(C)** and open states **(D)** generated based on the structures of the zebrafish P2X4.1R (4DW0 and 4DW1, respectively). The overall architecture of single P2XR subunit is analogous to the shape of a leaping dolphin, with the extracellular and TM domains akin to the body and the tail, respectively. The different domains are shown in colors; tail in green, lower body in cyan, upper body in blue, head in purple, dorsal fin in orange, right flipper in red, and left flipper in yellow (the same color scheme as used in Figure 1). The five conserved disulfide bonds are shown in white. The dotted circles **(C)** highlight the major differences in the human P2X7R subunit compared with the zebrafish P2X4.1R subunit, and the solid circles the major differences between the closed and open states of the same receptors **(B,D)**.

The structures in the closed and ATP-bound open states of the zebrafish P2X4.1R in truncated forms consisting of the extracellular and TM domains have been recently solved at 2.9 and 2.8 Å, respectively (Hattori and Gouaux, 2012). The overall architecture of the single subunit without the intracellular N- and C-terminal domains has been imaginatively described to resemble the shape of a dolphin leaping from the ocean (Figures 2A,B), with the extracellular domain akin to the body and the TM domain to the tail submerged in the water (Kawate et al., 2009; Hattori and Gouaux, 2012). The structure in the closed state contains eight α-helices (α1–α8) and fourteen β-strands (β1–β14) connected by loops with less defined structures (Figure 2A). In the open state, the α7 helix is absent in the lower body domain and a short α-helix appears in the head domain (Figure 2B). The multiple anti-parallel β-strands in the upper and lower body domains form “connecting rods” (Browne et al., 2010), which link the extracellular and TM domains.

The extracellular and TM domains of the zebrafish P2X4.1R and mammalian P2X7Rs show substantial sequence relatedness (Figure 1), enabling structural models based on the former receptor to provide meaningful insights into the molecular basis for the functional properties of the latter receptors. Because of insertions and deletions in the sequence relative to that of the zebrafish receptor, the models of the human P2X7R subunit in the closed and open states (Figures 2C,D) show several differences from the corresponding zebrafish P2X4.1R subunit structures (Figures 2A,B), including the presence of a much longer loop connecting the β3 and β4 strands in the upper body domain and loss of the α4 helix in the dorsal fin domain (Figures 2C,D).

**Figure 3:**
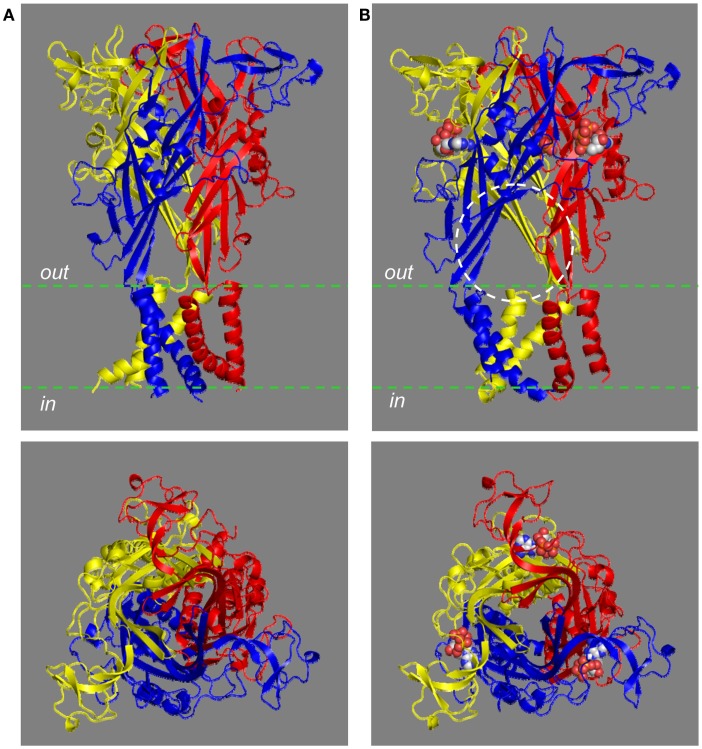
**The chalice-like structural models of the human P2X7R**. The structural models of the trimeric human P2X7R in the closed **(A)** and ATP-bound open state **(B)**, based on the structures of the zebrafish P2X4.1R (4DW0 and 4DW1, respectively), are viewed parallel to the plasma membrane (top) or from the extracellular side of the membrane (bottom). Each subunit is shown in a different color. Three ATP molecules shown in space filling representation bind to the three inter-subunit interfaces. The circle [top in **(B)**] shows one of the three lateral fenestrations for ions to enter or exit from the transmembrane ion-conducting pathway in the open state.

The trimeric P2XR has a chalice-like shape (Kawate et al., 2009; Hattori and Gouaux, 2012). In the structural models of the homo-trimeric P2X7R in both the closed and open states, the three subunits are positioned in a threefold symmetry around an axis perpendicular to the plasma membrane and running through the center of the receptor (Figure 3). The three extracellular domains are tightly intertwined, making contacts at several points. The TM domain is made of six helices, two from each of the three subunits; the three TM1 helices are located at the periphery, and the three TM2 helices in the center and, when viewed parallel to the plasma membrane, they all tilt at ∼45° away from an axis perpendicular to the plasma membrane. Comparison of the closed and open state structures indicates that the upper body domains remain relatively static, whereas the other extracellular domains of the protein such as the head and left flipper domains and the TM domain undergo apparent conformational changes during receptor activation (Figures 2B,D and 3).

### The core residues involved in ATP-binding

Extracellular ATP is the only physiological agonist for the P2XRs, but molecular cloning and sequence analyses indicate absence of known ATP-binding consensus sequences. Detailed analysis of the concentration-receptor ion channel activation curves and the single channel activation kinetics suggests binding of three ATP molecules to the mammalian P2XR or presence of three ATP-binding sites (Bean, 1990; Ding and Sachs, 1999; Jiang et al., 2003). It is worth mentioning that recent studies show that occupancy of two of the three ATP-binding sites is sufficient for receptor activation (Wilkinson et al., 2006; Stelmashenko et al., 2012). Extensive site-directed mutagenesis and functional studies have been conducted, leading to identification of a subset of conserved hydrophilic residues in the extracellular domain that are key determinants for activation of the mammalian P2XRs by ATP and structurally related agonists (Evans, 2009, 2010; Browne et al., 2010). Further investigations suggest that these residues are from two neighboring subunits and that ATP binds at the subunit interface (Wilkinson et al., 2006; Marquez-Klaka et al., 2007, 2009). Despite all the efforts, the structural basis for ATP-binding remained hypothetical until the groundbreaking progress in solving the atomic structure of the zebrafish P2X4.1R in complex with ATP (Hattori and Gouaux, 2012). This ATP-bound structure reveals for the first time the ATP-binding site of the P2XR, and nicely endorses the ATP-binding features inferred from mutagenesis studies of the mammalian P2XRs. The three ATP-binding pockets are readily visible at the three subunit interfaces (Figure 3B). The ATP-binding site is situated at the apex of the ATP-binding pocket and consists of 10 residues from two adjacent subunits in the zebrafish P2X4.1R; one subunit contributes four hydrophilic residues (Lys70, Lys72, Thr189, and Lys193) and a further three hydrophobic residues (Leu191, Leu217, and Ile232) while the complementary subunit offers three hydrophilic residues (Asn296, Arg298, and Lys316) (Hattori and Gouaux, 2012). The seven hydrophilic residues are almost completely conserved in the mammalian P2XRs, and correspond to Lys64, Lys66, Thr189, Lys193, Asn292, Arg294, and Lys311 in the human P2X7R (Figure 1). They have been shown to be critical in activation of the mammalian P2XRs including the P2X7Rs (Worthington et al., 2002; Wilkinson et al., 2006; Adriouch et al., 2008; Schwarz et al., 2009; Browne et al., 2010). The three hydrophobic residues are found in some but not all the mammalian P2X1R–P2X6R, the hydrophobicity, which is important in interaction with ATP, is nevertheless highly preserved at the equivalent three positions (Kawate et al., 2009). The mammalian P2X7Rs contain Leu191 and Ile228, equivalent to Leu191 and Ile232 in the zebrafish P2X4.1R, but apparently lack a residue corresponding to Leu217 (Figure 1).

**Figure 4:**
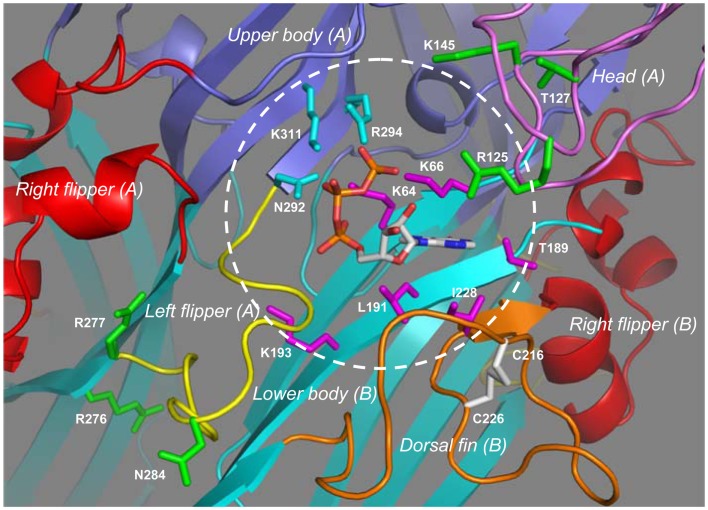
**The inter-subunit ATP-binding site in the human P2X7R**. Binding of ATP to the inter-subunit site in the human P2X7R in the open state involves nine residues from two adjacent subunits. The subunit-B contributes four hydrophilic residues (Lys64, Lys66, Thr187, and Lys197) and a further two hydrophobic residues (Leu191 and Ile228) shown in magenta, and the complementary subunit-A provides another three hydrophilic residues (Gln292, Arg294, and Lys311) in cyan. The circle shows the approximate boundary of the ATP-binding site. Changes of residues shown in green alter the agonist sensitivity or ion channel gating of the mammalian P2X7Rs with the exception of Arg125, which is the ADP-ribosylation site.

Figure 4 shows the ATP-binding site in the structural model of the human P2X7R in the ATP-bound open state. ATP adopts a *U*-shaped conformation. The triphosphate moiety and specifically the β- and γ-phosphate groups are partially exposed. Lys64 in the lower body in one subunit (referred to as subunit-B in Figure 4) and particularly its side-chain ${\text{NH}}_{\text{3}}^{\text{+}}$ group is situated close to the center of the triphosphate moiety, and forms hydrogen bonds with all three phosphate groups. The side chains of Lys66 and Lys193, also located in the lower body of the same subunit, interact with the γ- and α-phosphate groups, respectively; the interaction of Lys193 with the α-phosphate group is predicted to be mediated by a water molecule (not shown in Figure 4). The side chains of Asn292 and Lys311 and those of Arg294 and Lys311 in the upper body of the complementary subunit (subunit-A in Figure 4) interact via hydrogen bonds with the β- and γ-phosphate groups, respectively. The adenine base forms hydrogen bonds with the side chain of Thr189 and the main-chain carbonyl oxygen atoms of Lys66 and Thr189 in the lower body (subunit-B), and also makes hydrophobic interactions with Leu191 in the lower body and Ile228 in the dorsal fin in the same subunit. A recent study has shown a critical role for Leu186 in the rat P2X2R, equivalent to Leu191 in the human P2X7R, in recognizing the adenine base (Jiang et al., 2011). Mutation of Leu191 to proline by NS-SNP in the human P2X7R impaired the receptor function (Roger et al., 2010b). The hydroxyl groups at positions 2′ and 3′ of the ribose ring face outwards, a situation suggests that the ATP-binding site would readily tolerate attachment of a bulky moiety to these positions. Consistent with this hypothesis, BzATP acts as a potent agonist at all the P2XRs. As mentioned above, a residue corresponding to Leu217 in the dorsal fin domain of the zebrafish P2X4.1R, which in the latter specifically interacts with the ribose ring (Hattori and Gouaux, 2012), is lacking in the mammalian P2X7Rs. It is thus tempting to speculate that loss of this residue or a difference in the local structure of this region of the receptor is responsible for the low ATP sensitivity and the higher potency of BzATP than ATP, the hallmark pharmacological properties of the P2X7Rs. Interestingly, the corresponding region of the dorsal fin domain is variable in sequence and length, and does contain a hydrophobic residue, Ile214 in the human P2X7R (Figure 1). It remains unclear whether this residue participates in the interaction with the ribose ring.

### Contributions from other parts or residues surrounding the ATP-binding site

The structural models also give some insights into the roles of parts or residues surrounding the ATP-binding site in determining the functional properties of the mammalian P2X7Rs. Measurements of BzATP-induced ionic currents showed that the charge-neutralizing K145A mutation in the rat P2X7R reduced the BzATP sensitivity (Liu et al., 2008). This residue is located in the head domain, adjacent to the ATP-binding site (Figure 4). The positive charge of Lys145 is present in all the mammalian P2X7Rs (Figure 1) and may facilitate agonist access or binding to the ATP-binding site. In contrast, the charge-neutralizing R276A mutation in the mouse P2X7R increased the sensitivity to ATP and BzATP, as revealed by agonist-induced increases in the cytosolic Ca^2+^ level and YO-PRO-1 uptake, and also slowed the open channel deactivation (Adriouch et al., 2009). However, the charge-conserving R276K mutation and, similarly the R277K mutation, enhanced ATP-induced increases in the cytosolic Ca^2+^ level and YO-PRO-1 uptake without affecting the agonist sensitivity (Adriouch et al., 2008, 2009). The NS-SNP R276H mutation increased (or H276R reduced as described in the original study) ATP-induced ethidium uptake (Stokes et al., 2010). This pair of positively charged residues (R276 and R277 in the human P2X7R) is conserved in the mammalian P2X7Rs (Figure 1) and also present in some other P2XRs (Kawate et al., 2009). The structural models place them in the lower body domain and away from the ATP-binding site (Figure 4). The mutational effects together with their structural location suggest that these two residues are likely involved in conformational changes accompanying ion channel gating or receptor activation/deactivation.

The mammalian P2X7Rs exhibit striking differences in their agonist sensitivity. Based on determination of the concentrations required to induce 50% of the maximal ionic currents (EC_50_), the rat P2X7R exhibits 3 ∼ 10- and 30 ∼ 100-fold higher sensitivity to ATP, and BzATP, respectively, than the human (Rassendren et al., 1997b; Bradley et al., 2011a) and mouse P2X7Rs (Young et al., 2007). Substitution of Val154-Asn183 in the human P2X7R with the corresponding segment from the rat P2X7R increased the sensitivity to both ATP and BzATP (Michel et al., 2008). Replacement of the entire extracellular domain (Ser47-Val334) with that of the rat P2X7R caused the mouse P2X7R to show virtually the same agonist sensitivity as the rat P2X7R, indicating that the molecular determinants for the species difference in the agonist sensitivity reside in the extracellular domain. The sensitivity to ATP and BzATP of a mouse P2X7R, in which two small segments, Gln115-Ile136 and Tyr282-Phe288, were both substituted by the corresponding ones of the rat P2X7R, was similar to that of the rat P2X7R (Young et al., 2007). In the human P2X7R structural models, Glu115-Lys136 (corresponding to Gln115-Lys136 in the mouse P2X7R) and the N-terminal half (Val154-Cys168) of Val154-Asn183 contribute to the head domain, the C-terminal half (Pro169-Asn183) forms the loop connecting the head domain and the α3 helix in the right flipper domain, and Thr282-Phe288 (equivalent to Tyr282-Phe288 in the mouse P2X7R) is in the left flipper domain. These regions represent the key structural components that embrace the ATP-binding site (Figure 4) and, as discussed below, undergo significant conformational changes during receptor activation. Characterization of the mouse P2X7R carrying individual residues that are only present in the rat P2X7R, has identified Asn284, and Lys127 in pair with Asn284, as critical determinants for the higher sensitivity of the rat P2X7R to ATP and BzATP, respectively (Young et al., 2007). Position 284 (occupied by Asn284 in the human P2X7R; Figure 4) is situated at the tip of the left flipper or the bottom half of the ATP-binding pocket; the negative charge of Asp284 in the mouse P2X7R may hinder agonist access to the ATP-binding site, agonist-induced conformational changes or both. Position 127 (occupied by Thr127 in the human P2X7R) has been proposed to interact with BzATP (Young, 2010). However, this residue is located on the outer edge of the top half of the ATP-binding pocket and distant from the ATP-binding site (Figure 4) and thus the positive charge of Lys127 in the rat P2X7R is more likely to increase agonist accessibility to the ATP-binding site and/or assist agonist-induced conformational changes.

The mouse P2X7R can be activated by extracellular nicotinamide adenine dinucleotide (NAD) independently of, or in synergy with, ATP via ADP-ribosylation of the P2X7R protein catalyzed by ADP-ribosyltransferase 2 ecto-enzymes (Seman et al., 2003; Adriouch et al., 2008; Hong et al., 2009; Schwarz et al., 2012; Xu et al., 2012). Arg125 in the extracellular domain has been identified by site-directed mutagenesis as the site for ADP-ribosylation (Adriouch et al., 2008). ADP is known to poorly activate the mammalian P2XRs including the mouse P2X7R (North and Surprenant, 2000). The original study nonetheless hypothesized that Arg125 is located in the close vicinity of the putative ATP-binding site and ADP-ribosylation enables binding of the ADP or ADP-ribose moiety to the ATP-binding site and thereby activation of the receptor (Adriouch et al., 2008). The corresponding Arg125 in the human P2X7R is in the head domain and indeed, as predicted, projects toward the ATP-binding site from the tip of the top half of the ATP-binding pocket (Figure 4), supporting the hypothesis that ADP-ribose binds to the same site as ATP (Young, 2010). Arg276 is not an ADP-ribosylation site, but the R276K mutation significantly increased the NAD sensitivity of the mouse P2X7R (Hong et al., 2009; Schwarz et al., 2012). As discussed above, this residue is located in the lower body domain and away from the ATP-binding site (Figure 4) and thus the mutational effect on the NAD sensitivity is likely to result from facilitation of conformational changes leading to receptor activation.

Thus studies so far have provided clear evidence to support the idea that the residues surrounding the ATP-binding site are also important in determining the agonist sensitivity of the mammalian P2X7Rs, including the species differences in the agonist sensitivity. Evidently, more studies are required to provide a better and mechanistic understanding of their contributions. It is interesting and also important to know whether the P2XR7-specific regions or residues that surround the ATP-binding site are crucial in determining the high potency and selectivity of P2X7R antagonists and particularly those of competitive antagonists (Jiang et al., 2012).

### The small cation-conducting pathway

**Figure 5:**
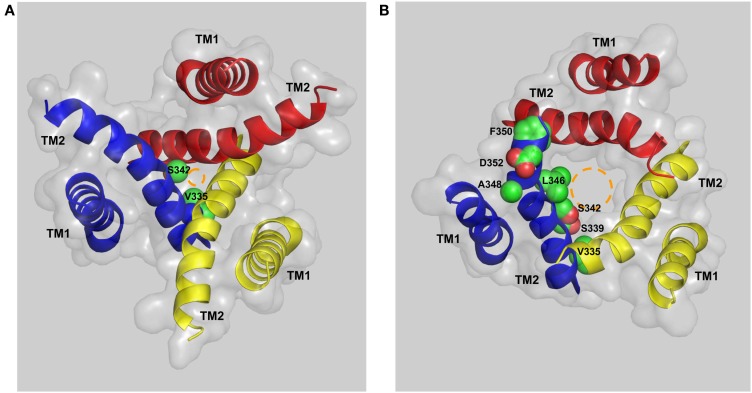
**The transmembrane ion-conducting pathway in the human P2X7R**. The arrangements of the transmembrane domains (TM1s and TM2s) in the structural models of the human P2X7R in the closed **(A)** and open states **(B)**, viewed from the intracellular side of the membrane. The three TM1 helices are located at the periphery and the three TM2 helices in the center form the ion-conducting pathway. Val335 and Ser342 represent the extracellular and intracellular ends of the gate that restricts the ion flow in the closed state **(A)**. Val335, Ser339, Ser342, Leu346, Ala348, Phe350, and Asp352 form the ion-conducting pathway in the open state **(B)**. The circles in brown illustrate the change in size of the ion-conducting pathway from the closed to the open state.

Activation of the mammalian P2X7Rs opens, within milliseconds, a transmembrane ion-conducting pathway selective for small cations such as Ca^2+^, Na^+^, and K^+^. In the structural model of the human P2X7R, the three TM2 helices, viewed from the cytoplasmic side of the plasma membrane, are seen to come into close proximity at the approximate center of the bilayer, forming a constriction, or physical gate that restricts ion flow in the closed state (Figure 5A). Val335 and Ser342 (corresponding to Leu340 and Ala347 in the zebrafish P2X4.1R) are located at the extracellular and intracellular ends of the gate, respectively. In the open state, Val335, Ser339, Ser342, Leu346, and Phe350 (equivalent to Leu340, Ala344, Ala347, Leu351, and Ile355 in the zebrafish P2X4.1R) represent the major structural components facing to the ion-conducting pathway (Figure 5B). The narrowest part of the open ion-conducting pathway in the zebrafish P2XR4.1 is defined by three Ala347 residues with their Cα atoms being 6.4 Å from the central axis (Hattori and Gouaux, 2012). The structural model predicts a very similar size for the narrowest part of the open ion-conducting pathway of the human P2X7R that is defined by the corresponding Ser342 residues (Figure 5B). The same subset of residues is expected to line the ion-conducting pathway in the other mammalian P2X7Rs with the exception of Phe350, which is replaced with cysteine in the rat and mouse P2X7Rs (Figure 1). Introduction of the F350C mutation in the human P2X7R however had no effect on the receptor function (Bradley et al., 2011a). As discussed below, the A348T mutation by NS-SNP in the human P2X7R, and the reciprocal T348A mutation in the rat P2X7R, significantly altered the P2X7R ion channel function. In the rat P2X7R, attachment of a positive charge at position 348 by the T348K mutation, neutralization of the negative charge at Asp352 by the D352N mutation or replacement with a positive-charge by the D352K mutation conferred a modest Cl^−^ permeability (Browne et al., 2013). Furthermore, mutation of the equivalent residues in the rat P2X2R, Ser345 and Asp349, changed single channel conductance (Cao et al., 2009). All these mutagenesis studies consistently suggest that the residues at positions 348 and 352 in the mammalian P2X7Rs and at equivalent positions in the other mammalian P2XRs are important parts of the transmembrane ion-conducting pathway when the receptors are in the open state.

The structure of the zebrafish P2X4.1R in the open state (Hattori and Gouaux, 2012) and the analysis of cysteine accessibility in the mammalian P2XRs (Kawate et al., 2011; Samways et al., 2011; Jiang et al., 2012; Roberts et al., 2012) provide consistent evidence in support of the notion that the three lateral fenestrations, located just above the plasma membrane and linking to the extracellular vestibule, act as the pathways for ions to enter or exit the aforementioned transmembrane ion-conducting pathway (Figure 3B). The lateral fenestrations contain negatively charged residues, Asp59 and Asp61 in the zebrafish P2X4.1R (corresponding to Arg53 and Glu55 in the human P2X7R; highlighted in blue in Figure 1). However, charge-neutralization of the corresponding negatively charged residues, Glu56, and Asp58, in the human P2X4R did not alter the cation selectivity. Thus, the cation selectivity of the P2XR ion channels has been attributed to the negative electrostatic milieu of the extracellular vestibule (Samways et al., 2011; Hattori and Gouaux, 2012). However, the contributing residues in the mammalian P2XRs including the P2X7Rs remain to be identified.

### The ion channel gating mechanism

The significant differences in the structures between the closed and open states have given an unprecedented insight into the general mechanism underlying ATP-induced gating of the P2XR ion channels (Hattori and Gouaux, 2012). Occupation of the ATP-binding site by extracellular ATP promotes lowering of the head domain in one subunit and elevation of the dorsal fin domain in the adjacent subunit to tighten the ATP-binding pocket and simultaneously causes a downward movement of the left flipper domain. Such conformational changes induce outward movements of the lower body domain or the “connecting rods” which in turn lead to enlargement of the lateral fenestrations and extracellular vestibule and to anticlockwise rotation of the TM helices around an axis perpendicular to the plasma membrane and their tilting away from an axis parallel to the plasma membrane. The iris-like movements of the TM helices cause the transition of the ion-conducting pathway from the closed to the open state (Figure 5). Such a gating mechanism is in principle supported by studies of the mammalian P2XRs (Cao et al., 2007, 2009; Browne et al., 2010, 2013; Li et al., 2010; Kawate et al., 2011; Jiang et al., 2012, 2013).

### The large pore and its enigmatic mechanism of formation

It has been known for decades that activation of the formerly named P2Z receptor in immune cells (Cockcroft and Gomperts, 1979; Gordon, 1986), which was renamed the P2X7R after its molecular cloning (Surprenant et al., 1996), induces formation of a large pore over tens of seconds to minutes. Similar formation of large pores has been also described for the P2X2R, P2X4R, P2X2/3R, and P2X2/5R (Khakh et al., 1999; Virginio et al., 1999b; Compan et al., 2012). Two fundamentally different pore-forming mechanisms were proposed more than 10 years ago (North, 2002), but the evidence subsequently obtained has been conflicting and difficult to reconcile with a single mechanism.

The first mechanism entails recruitment of a distinctive pore-forming protein as a result of activation of the P2X7R. The widely expressed pannexin-1 channel has attracted great attention, because pharmacological inhibition using the channel inhibitor, carbenoxolone, and the mimetic inhibitory peptide, ^10^Panx, or selective knockdown of the pannexin-1 protein expression using small interference RNA, strongly suppressed BzATP-induced YO-PRO-1 uptake without effect on BzATP-induced ionic currents in HEK293 cells heterologously expressing the rat P2X7R (Pelegrin and Surprenant, 2006). These findings have recently been recapitulated in peritoneal macrophage cells endogenously expressing the mouse P2X7R (Sorge et al., 2012); both carbenoxolone and ^10^Panx prevented BzATP-induced YO-PRO-1 uptake but did not alter BzATP-induced increases in the cytosolic Ca^2+^ level while, by contrast, brilliant blue G, a P2X7R antagonist, inhibited both BzATP-induced responses. Another recent study has reported that BzATP-induced little YO-PRO-1 uptake in astrocytes isolated from pannexin-1 transgenic knockout (KO) mice in contrast with the robust dye uptake in astrocytes from wild-type mice (Suadicani et al., 2012). These studies clearly support an indispensable role for pannexin-1 in P2X7R-dependent pore formation. Treatment of cells heterologously expressing the rat P2X7R or P2X2R with colchicine to disrupt the microtubule cytoskeleton reduced ATP-induced dye uptake without changing ATP-induced ionic currents, and similar differential effects were seen in mouse peritoneal macrophages endogenously expressing the mouse P2X7R (Marques-da-Silva et al., 2011). As discussed later, the NS-SNP P451L mutation in the C-terminus of the mouse P2X7R can also discriminate the two receptor functions in macrophage cells by selectively impairing the large pore formation without effect on the ion channel function. These observations are consistent with the idea that the large dye uptake pore is different from the small ion-permeable channel, although both depend on the P2XR activation (Pelegrin, 2011). However, other studies strongly argue against the role of pannexin-1; for example, there was no significant change in ATP-induced YO-PRO-1 uptake in macrophage cells isolated from another pannexin-1 KO mouse strain (Qu et al., 2011). Pharmacological inhibition of the pannexin-1 channel failed to alter the large pore formation induced by activation of the P2X2R heterologously expressed in HEK293 cells (Chaumont and Khakh, 2008) or the P2X4R endogenously expressed in microglia (Bernier et al., 2012).

In the second mechanism, the large pore results from progressive dilatation of the small ion-conducting pathway. The hypothesis that the large pore represents an intrinsic functional property of the P2X7R more readily explains the remarkable differences in the large pore-forming capacity and dynamics upon activation of P2X7Rs with a diversity of structural differences. Thus, the rat P2X7R still functioned as a ligand-gated ion channel following deletion of most of the C-terminal domain but failed to induce YO-PRO-1 uptake (Surprenant et al., 1996). Similar functional properties have been described for the human P2X7B alternative splicing isoform, which has a very short C-terminal domain due to replacement of the sequence comprising the last 249 residues of the full-length protein (P2X7A) with an alternative 18 residue sequence, whereas the heteromeric P2X7R formed by the P2X7A and P2X7B subunits exhibits a greater capacity of forming the larger pore than the homomeric P2X7A receptor (Adinolfi et al., 2010). The mouse P2X7k alternative splicing isoform, with a different N-terminus and intracellular half of the TM1 helix, can induce large pore formation with faster kinetics (Nicke et al., 2009; Xu et al., 2012). In particular, substitution with tyrosine of the highly conserved Gly345 residue in the rat P2X7R corresponding to Gly350 in the zebrafish P2X4.1R, located in the TM2 helix (Figure 1) and on the intracellular side of the gate (Figure 5), caused complete loss of ATP-induced dye uptake without effect on ATP-induced increases in the cytosolic Ca^2+^ level (Monif et al., 2009). Mutations of the corresponding glycine residues in the P2X2R (Gly342) and P2X4R (Gly347) (Khakh et al., 1999; Virginio et al., 1999b) or of residues in the C-terminal domain of the P2X2Rs (Eickhorst et al., 2002) resulted in diverse and contrasting effects on the large pore formation. A recent study has provided compelling evidence to show that the rat P2X7R ion channel carrying G345C or T348C mutations was able to pass large molecules with sizes up to 14 Å (Browne et al., 2013). The acid-sensing ion channels (ASICs), despite exhibiting no significant sequence relatedness, have virtually the same membrane topology and subunit stoichiometry as the P2XRs, and in fact, comparison of the atomic structures of the chicken ASIC and zebrafish P2X4.1R indicates a similar architecture of the ion-conducting pathways (Gonzales et al., 2009; Browne et al., 2010). As revealed in a recent study, the ASIC channel has two distinctive open states of the ion-conducting pathway; one narrow open and Na^+^-selective state at low extracellular pH, and one wide open and non-selective state at high extracellular pH (Baconguis and Gouaux, 2012). Thus, it is conceivable that agonist ligation of the P2XRs induces initial opening of the small cation-conducting pathway which subsequently expands and forms the large dye uptake pore.

In summary, the molecular basis for the large pore persistently remains elusive. P2X7R-dependent large pore formation nonetheless acts as a signaling mechanism that plays a crucial role in thymocyte cell death induced by ATP (Le Stunff et al., 2004) and microglial activation and proliferation due to tonic receptor activation (Monif et al., 2009) and also, as have been shown in recent studies, in a number of diseases, including chronic pain (Sorge et al., 2012), pulmonary fibrosis (Riteau et al., 2010), inflammatory bowel diseases (Gulbransen et al., 2012), and osteoporsis (Syberg et al., 2012). More studies are required to elucidate the large pore-forming mechanisms as such information is obviously necessary for a better understanding of the physiological and pathophysiological roles of the mammalian P2X7Rs and identification of more specific intervention targets for development of therapeutics.

## NS-SNP Insights into Mechanisms for P2X7R Functions and Diseases

### Implications in diseases

Non-synonymous SNPs in the *P2RX7* gene have been identified in patients with an increasing number and variety of conditions, including chronic lymphocytic leukemia (CLL) (Thunberg et al., 2002; Wiley et al., 2002; Starczynski et al., 2003; Zhang et al., 2003; Dao-Ung et al., 2004; Nückel et al., 2004; Chong et al., 2010), bipolar disorder (BP), major depressive disorder (MDD), and anxiety disorders (Barden et al., 2006; Lucae et al., 2006; Erhardt et al., 2007; Green et al., 2009; Grigoroiu-Serbanescu et al., 2009; Hejjas et al., 2009; McQuillin et al., 2009), pulmonary tuberculosis (Franco-Martinez et al., 2006; Nino-Moreno et al., 2007), osteoporosis in post-menopausal women (Ohlendorff et al., 2007; Gartland et al., 2012; Jørgensen et al., 2012) and fracture patients (Husted et al., 2013; Wesselius et al., 2013), multiple sclerosis (MS) (Oyanguren-Desez et al., 2011), systemic lupus erythematosus (SLE) and rheumatoid arthritis (RA) (Portales-Cervantes et al., 2012), ischemic stroke (IS) and ischemic heart disease (IHD) in smokers (Gidlof et al., 2012), chronic inflammatory and neuropathic pain (Sorge et al., 2012). More importantly, genetic linkage studies have gathered evidence to support an association of NS-SNPs with altered disease susceptibility, including A76V with MS (Oyanguren-Desez et al., 2011); G150R with osteoporosis in fracture patients (Husted et al., 2013; Wesselius et al., 2013); H155Y and R270H with chronic inflammatory pain (Sorge et al., 2012); R307Q with osteoporosis in post-menopausal women (Gartland et al., 2012; Jørgensen et al., 2012); A348T with anxiety disorders (Erhardt et al., 2007) and osteoporosis in post-menopausal women (Jørgensen et al., 2012) and fracture patients (Husted et al., 2013); R460Q with BP and MDD (Barden et al., 2006; Lucae et al., 2006; Erhardt et al., 2007; Hejjas et al., 2009; McQuillin et al., 2009; but, see Green et al., 2009; Grigoroiu-Serbanescu et al., 2009) and osteoporosis in post-menopausal women (Jørgensen et al., 2012) and fracture patients (Husted et al., 2013; Wesselius et al., 2013); E496A with CLL (Thunberg et al., 2002; Wiley et al., 2002; but, see Starczynski et al., 2003; Zhang et al., 2003; Nückel et al., 2004), tuberculosis (Nino-Moreno et al., 2007), IS, IHD (Gidlof et al., 2012) and osteoporosis in post-menopausal women (Ohlendorff et al., 2007; Jørgensen et al., 2012) and fracture patients (Husted et al., 2013; Wesselius et al., 2013); I568N with osteoporosis in post-menopausal women (Ohlendorff et al., 2007; Jørgensen et al., 2012). The P451L NS-SNP in the mouse *p2rx7* gene is associated with neuropathic pain (Sorge et al., 2012) and also with osteoporosis (Syberg et al., 2012).

### Effects on P2X7R functions

A small number of NS-SNP mutations in the human P2X7Rs have been examined so far in terms of their effects on the ion channel function, as measured by agonist-induced ionic currents, increases in the cytosolic Ca^2+^ level or preloaded Rb^+^ efflux, and the large pore function, as measured by agonist-evoked dye uptake. Agonist-induced increases in the cytosolic Ca^2+^ level and dye uptake in lymphocytes carrying the H155Y mutation were significantly increased, indicating expression of a hyper-functional mutant receptor (Cabrini et al., 2005; Portales-Cervantes et al., 2012). Similarly, the A348T mutation increased (or reduced by the T348A mutation described in the original study) agonist-induced dye uptake (Denlinger et al., 2006). In contrast, agonist-induced dye uptake levels in human lymphocyte, monocyte, and macrophage cells carrying the T357S mutation were decreased, indicating that the T357S mutant receptor was hypo-functional (Cabrini et al., 2005; Shemon et al., 2006). Expression of the R307Q mutant receptor in human monocyte, macrophage, lymphocyte, and natural killer (NK) cells resulted in complete loss of agonist-induced Rb^+^ efflux and dye uptake (Gu et al., 2004). Agonist-induced dye uptake was strongly impaired or completely lost in human monocyte, macrophage, lymphocyte, and NK cells harboring the P2X7R with the E496A or I568N mutations (Gu et al., 2001; Wiley et al., 2002, 2003; Sluyter et al., 2004; Denlinger et al., 2005).

**Figure 6:**
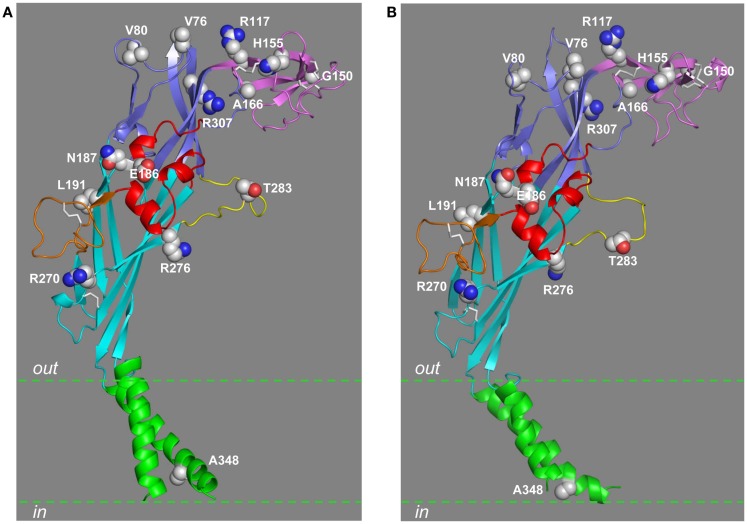
**Location of residues mutated by NS-SNPs in the extracellular and TM domains**. The residues in the extracellular and TM2 domains that are mutated by NS-SNPs are shown in the structural models of the human P2X7R subunit in the closed **(A)** and open states **(B)**. The same color scheme is used to indicate the different domains as in other figures. The residue at position 283 is mutated by NS-SNP in the mouse P2X7R.

The above six and a further 12 more NS-SNP mutations in the human P2X7R have been characterized in more detail upon heterologous expression. Twelve of them are located in the extracellular domain, including V76A, V80M, R117W, G150R, H155Y, A166G, E186K, N187D, L191A, R270H, R276H, and R307Q (Figures 1 and 6), which all significantly altered the receptor function except for V80M. G150R, E186K, R276H, and R307Q almost completely abolished, and V76A, R117W, N187D, R270H, and L191P reduced, whereas H155Y and A166G (or A76V and H270R reported in some studies) enhanced, agonist-evoked ionic currents and/or dye uptake without altering the agonist sensitivity except for N187D and possibly A166G (Gu et al., 2004; Cabrini et al., 2005; Chong et al., 2010; Roger et al., 2010b; Stokes et al., 2010; Sun et al., 2010; Bradley et al., 2011a; Oyanguren-Desez et al., 2011). The N187D mutation increased the agonist EC_50_ value by threefold (Chong et al., 2010). The A348T mutation in the TM2 helix (Figures 1 and 6) increased agonist-induced ionic currents and dye uptake without altering the agonist sensitivity (Cabrini et al., 2005; Roger et al., 2010b; Bradley et al., 2011a). The other five NS-SNP mutations, T357S, Q460R, E496A, H521Q, and I568N, are located in the cytoplasmic C-terminal domain. The T357S mutation in the proximal part of this domain reduced agonist-induced ionic currents and dye uptake without altering the agonist sensitivity (Shemon et al., 2006; Roger et al., 2010b). Both the Q460R and H521Q mutations had no effect on agonist-induced ionic currents, increases in the cytosolic Ca^2+^ level, dye uptake, and agonist sensitivity (Cabrini et al., 2005; Roger et al., 2010b). A very modest but significant reduction in agonist-induced dye uptake by the Q460R mutation was reported in another study (Stokes et al., 2010). The E496A mutation has been subject to scrutiny by several groups. Two earlier independent studies reported rather different effects on the ion channel function; the E496A mutant and wild-type receptors mediated similar agonist-induced ionic currents (Boldt et al., 2003) but the mutant receptor-mediated agonist-induced Ca^2+^ influx was largely lost (Cabrini et al., 2005). A subsequent study comparing ATP-induced ionic currents and dye uptake under the same experimental conditions showed that the E496A mutation strongly impaired both the ion channel and dye uptake pore functions to a similar degree without effect on the EC_50_ value for ATP (Roger et al., 2010b). Expression of the I568N mutant receptor resulted in no agonist-induced ionic current and dye uptake (Roger et al., 2010b).

A few human P2X7R variants carry multiple NS-SNP mutations (Stokes et al., 2010; Sun et al., 2010; Oyanguren-Desez et al., 2011). For example, the P2X7R-2 variant contains H270R and A348T mutations, and the P2X7R-4 variant has H155Y, H270R, A348T, and Q460R mutations (Stokes et al., 2010). These variants in human monocytes and erythrocytes mediated greater agonist-induced Rb^+^ efflux and/or dye uptake. Consistently, these variants in heterologous expression cells also exhibited larger agonist-induced ionic currents and dye uptake with a similar agonist sensitivity.

Characterizations of the NS-SNP mutations in the human P2X7R have shed new light on previously unidentified molecular determinants for the functions of the mammalian P2X7R and other P2XRs. Among the 12 extracellular residues subject to NS-SNP mutation (Figure 1), Gly150 and Arg307 are completely conserved and Gln187 is highly conserved in the mammalian P2XR family (Kawate et al., 2009). Ala166, Glu186, Leu191, and Arg276 are found in all mammalian P2X7Rs and also in some other mammalian P2XRs (Kawate et al., 2009). The prominent alterations in receptor functions by NS-SNP mutation of these residues indicate that they are crucial for the mammalian P2XR functions. Val76, Arg117, His155, and Arg270 are present in some mammalian P2X7Rs including the human P2X7R (Figure 1). The human P2X7R carrying the V76A, R117W, H155Y, or R270H mutations exhibited significant changes in receptor functions, suggesting that Val76, Arg117, His155, and Arg270 residues, albeit less conserved, are also important for the P2X7Rs. Ala348 is only present in the human P2X7R (Figure 1), and the A348T mutation resulted in a hyper-functional receptor, supporting a functional role for this residue. The hypo-functional phenotypes by NS-SNP mutation of Thr357, Glu496, and Ile568 indicate the importance of these residues in the C-terminal domain of all the mammalian P2X7Rs (Figure 1).

So far four NS-SNP mutations in the mouse P2X7R have been studied, including F11L in the N-terminus, A221T and M283T in the extracellular domain, and P451L in the C-terminus (Figure 1) (Adriouch et al., 2002; Le Stunff et al., 2004; Young et al., 2006, 2007). Phe11, Ala221, and Met283 are present in the mouse P2X7R sequence encoded by the cDNA initially cloned from NTW8 microglial cells, whereas Leu11, Thr221, and Thr283 are found in the P2X7R expressed in C57BL/6 and Balb/c mice (Young et al., 2006). Both receptors exhibit a similar agonist sensitivity, but agonist-induced ionic currents mediated by the receptor carrying Leu11, Thr221, and Thr283 were ≥8-fold larger than those by the receptor carrying Phe11, Ala221, and Met283 (Young et al., 2006). Similarly, agonist-induced dye uptake was robust in cells expressing the former receptor but was small or barely detectable in cells expressing the latter receptor. Additional efforts of swapping individual residues have identified the residue at position 283 as the key determinant (Young et al., 2006). The mouse P2X7R ion channels carrying Pro451 or Leu451 exhibited the same functional properties including agonist sensitivity, but the P451L mutation strongly impaired or completely abolished the ability of the receptor to induce large pore formation (Young et al., 2006; Sorge et al., 2012; Xu et al., 2012), suggesting a preferential role for the residue at position 451 in the pore-forming process.

### Structural insights into molecular mechanisms for the NS-SNP effects

It is known from the discussion above that Leu191 in the lower body domain is one of the three hydrophobic residues in the ATP-binding site and mediates hydrophobic interactions with the adenine ring (Figures 4 and 6). Thus, the L191P mutation conferred the hypo-functional phenotype most likely by impairing the hydrophobic interactions with ATP as proposed in the original study (Roger et al., 2010b). Arg117, Gly150, His155, and Ala166 are all in the head domain, with Arg117 in the β5 strand, Gly150 immediately before the β6 strand, His155 in the loop between the β6 and β7 strands, and Ala166 in the β7 strand (Figure 6). Glu186 and Gln187 are in the α3 helix of the right flipper domain, and Arg270 and Arg276 are in the β12 strand leading toward the left flipper domain (Figure 6). Thr283 in the human P2X7R (corresponding to Met283 in the mouse P2X7R) is located in the left flipper domain (Figure 6). These residues are within the above-mentioned structural components surrounding the ATP-binding site (Figure 4) and undergo conformational changes during receptor activation. The NS-SNP mutations are likely to alter the local structure and thereby the conformational changes. Finally, both Val76 and Arg307 are located in the upper body domain, with Val76 in the highly variable loop connecting the β3 and β4 strands and Arg307 in the N-terminus of the β14 strand (Figure 6). The upper body domain in the trimeric zebrafish P2X4.1R form “a rigid scaffold” as they show little mobility during receptor activation (Hattori and Gouaux, 2012). Interestingly, Arg312 in the structure of the zebrafish P2X4.1R in both the closed and ATP-bound open states, corresponding to Arg307 in the human P2X7R (Figure 1), is involved in a network of hydrogen-bonding and salt-bridge interactions with Gln114 in the β5 strand in the same chain and also Asp88 and Asp91 in the α2 helix in the adjacent subunit (Figure 1). These residues are highly conserved in the mammalian P2XRs including the P2X7R, corresponding to Gln114, Asp89, and Asp92 in the human P2X7R. *In silico* mutation of Arg312 to glutamine or to another positively charged lysine residue leads to apparent loss of most of these interactions, and thus it is tempting to hypothesize that disruption of such interactions may be responsible for the loss of function associated with the R307Q NS-SNP mutation in the human P2X7R (Roger et al., 2010b) and the R307K mutation in the mouse P2X7R (Adriouch et al., 2008), and mutation of the corresponding residues in other mammalian P2XRs (Jiang et al., 2000; Browne et al., 2010; Evans, 2010). Ala348 is located in the TM2 helix (Figure 6) and, as discussed below, there is evidence suggesting that the A348T mutation influences the ion channel gating and large pore formation. Currently, there is no structural information for the residues mutated by NS-SNPs in the C-terminus (Thr357, Pro451, Glu496, and Ile568). It is worthwhile pointing out that some of the NS-SNP mutations, such as I568N (Wiley et al., 2003) and H155Y mutations as discussed below (Bradley et al., 2011a), can mainly or additionally alter the expression of the receptor at the cell surface.

### Molecular mechanisms determining species differences in receptor functions

H155Y and A348T are outstanding among all the NS-SNP mutations identified so far in the human P2X7R, as they replace the residues in the human P2X7R with the ones in the rat and other mammalian P2X7Rs (Figure 1). Agonist-induced ionic current and dye uptake mediated by the human P2X7R are significantly smaller than those by the rat P2X7R (Rassendren et al., 1997b; Bradley et al., 2011a). Both H155Y and A348T mutations gave rise to hyper-functional receptors mediating increased agonist-induced ionic currents and dye uptake, suggesting a role for the residues at these two positions in determining the difference in agonist-induced responses mediated by the human and rat P2X7Rs. Consistently with this idea, introduction of the reciprocal Y155H and T348A mutations in the rat P2X7R singly or together resulted in the opposing effects by reducing agonist-induced ionic currents (Bradley et al., 2011a). None of these mutations altered the agonist sensitivity. The amplitude of agonist-induced ionic currents is in principle governed by two distinctive mechanisms, that is, the single channel properties (the single channel conductance and opening probability) and the surface expression (the number of functional channels in the plasma membrane). To further elaborate their role, His155 and Phe348 in the human P2X7R were systematically replaced by residues bearing side chains differing in size, shape, or charge (Bradley et al., 2011a). All the mutant receptors exhibited a similar ATP sensitivity. None of the six substitutions introduced into the His155 position mimicked H155Y, and the mutational effects exhibited no clear relationship to the physiochemical properties of the side chain of the introduced residues, indicating that Tyr155 is unique in conferring the hyper-functional phenotype. In contrast, the effects of mutating Ala348 were inversely correlated with the volume of the residues; the residue with the smallest side chain conferred the greatest increase in the amplitude of ATP-induced ionic currents, and the ones with the larger side chains caused the greatest decrease, an effect that is anticipated if the residue at this position is involved in determining the single channel conductance and/or opening probability (Bradley et al., 2011a). Consistently with this hypothesis, recent studies, as discussed above, have presented compelling evidence to show that the residue at this position in the rat P2X7R and at the equivalent position in the rat P2X2R contributes to formation of the transmembrane pathways permeating both small ions and large organic molecules (Cao et al., 2009; Browne et al., 2013). Immunofluorescent confocal imaging and biotin-labeling assays have demonstrated that the H155Y mutation enhanced the surface expression of the human P2X7R and, conversely, the reciprocal Y155H mutation reduced the surface expression of the rat P2X7R. Such complementary effects were not observed for the A348T mutation in the human P2X7R and the T348A mutation in the rat P2X7R, both of which had negligible effects on the surface expression of the P2X7Rs (Bradley et al., 2011a). As already described above, the residue at position 155 is located in the loop connecting the β6 and β7 strands in the head domain, whereas the residue at position 348 is present in the TM2 helix and on the intracellular side of the ion channel gate (Figures 5 and 6). The contrasting mutational effects on the ion channel function and surface expression of the receptors are consistent with their structural locations and together support the conclusion that the residues at positions 155 and 348 contribute to the functional difference between the human and rat P2X7Rs by modulating the surface expression and single channel function, respectively (Bradley et al., 2011a).

The M283T and P451L mutations rendered the mouse P2X7R to be hyper-functional and hypo-functional, respectively. Thr283 and Pro451 are present in the human and other mammalian P2X7Rs except for the guinea pig P2X7R in which Thr283 is replaced by valine (Figure 1). Therefore, the residues at these two positions may also contribute to the species difference between the human and rodent P2X7Rs. Neither the M283T nor the P451L mutation altered the surface expression of the mouse P2X7R (Young et al., 2006, 2007; Sorge et al., 2012). The residues at position 283 is located in the left flipper domain that represents one of the structural components surrounding the ATP-binding site (Figure 4). Mutation of the residue at this position may fine-tune the conformational changes accompanying receptor activation. Position 451 is located in the middle of the cytoplasmic C-terminal sequences (Figure 1), for which no structural information is available. As discussed above, the residue at this position is critical for the ability to induce large pore formation but not for the ion channel function of the P2X7Rs, the reason for this remains elusive.

In summary, characterizations of the NS-SNPs in the human and rodent P2X7Rs that swap species-specific residues have revealed distinctive molecular mechanisms that determine the functional differences in mammalian P2X7Rs.

### P2X7R-mediated molecular mechanisms in diseases

The expression of the P2X7Rs in lymphocytes is well-documented. CLL is a hematopoietic malignant tumor resulting from excessive monoclonal expansion of B-lymphocytes and accumulation of CD5^+^ lymphocytes in the blood. The basal or tonic level of the P2X7Rs or activation at a low level is known to promote proliferation or growth of cells including lymphoid cells (Baricordi et al., 1999; Adinolfi et al., 2005). Expression of the P2X7R was greater in lymphocytes from CLL patients with the progressive variant of the disease than in lymphocytes from patients with the indolent or less severe variant, suggesting altered P2X7R expression and function to contribute to the pathogenesis and progression of CLL (Adinolfi et al., 2002). Strong stimulation of P2X7Rs can induce cell death, garnering the reputation of a cytolytic receptor (Surprenant et al., 1996; Virginio et al., 1999a). A recent study has investigated the role in tumorigenesis of the N187D mutation that was identified in human leukemic cells and resulted in a hypo-functional receptor with reduced agonist sensitivity as discussed above (Chong et al., 2010). The mutation enhanced the ability of the receptor to promote cell proliferation associated with the basal receptor activity, and reduced the ability of the receptor to mediate BzATP-evoked cell apoptosis. Relative to the tumors induced by cells heterologously expressing the wild-type receptor implanted in immune-deficient mice, the tumors induced by cells expressing the mutant receptor grew faster, the size and weight being larger and heavier, and the tumor tissues showed heightened angiogenesis and macrophage infiltration. Such a detailed study of the N187D NS-SNP mutation provides a new insight into how altered P2X7R expression and function leads to hematopoietic malignancies such as CLL.

Studies using transgenic KO mice and specific receptor antagonists have shown a critical role for the P2X7R in mediating chronic neuropathic and inflammatory pain in rodent animals, but the underlying receptor mechanisms remain less understood (Jiang, 2012). A recent study has reported that the mouse strains expressing the Pro451 allele encoding the wild-type receptor exhibited more intense mechanical allodynia induced by nerve injury than those expressing the Leu451 allele encoding the P451L mutant receptor (Sorge et al., 2012). BzATP-induced similar increases in the cytosolic Ca^2+^ level in macrophage cells from A/J mice expressing the Pro451 allele and from B10.D2 mice expressing the Leu451 allele, but induced large pore formation only in macrophage cells from the A/J mice. Furthermore, BzATP-induced large pore formation in macrophage cells from the A/J mice was strongly impaired following treatment with a peptide flanking Pro451 (Ser445-Gln455) of the mouse P2X7R and fused to the HIV-1 TAT domain (TAT-Pro451), but not with the TAT-peptide containing Leu451 (TAT-Leu451). Neither of the TAT-peptides altered BzATP-induced increases in the cytosolic Ca^2+^ level. Intravenous fusion of the TAT-P451 peptide into the A/J mice strongly inhibited allodynia induced by nerve injury and inflammatory allodynia induced by injecting complete Freund’s adjuvant in the hind paws. The same study has also examined a cohort of patients reporting chronic post-mastectomy pain; individuals carrying the Tyr155 allele encoding the hyper-functional H155Y mutant receptor suffered a higher level of pain compared to those with the alleles encoding the wild-type receptor and, by contrast, individuals with the His270 allele encoding the hypo-functional R270H mutant receptor experienced a lower level of pain. In addition, the R270H NS-SNP showed strong association with chronic inflammatory pain in a cohort of osteoarthritis patients. These findings indicate P2X7R-dependent large pore-forming function is critical in determining the sensitivity to chronic neuropathic and inflammatory pain.

P2X7 receptor is expressed in both osteoblasts and osteoclasts and plays a crucial role in regulating bone remodeling (Ke et al., 2003). As mentioned above, several NS-SNPs have been identified in osteoporosis or fracture patients. Recent genetic linkage analyses of separate cohorts of women with post-menopausal bone loss and fractures (Ohlendorff et al., 2007; Gartland et al., 2012; Jørgensen et al., 2012) and cohorts of fracture patients including both women and men (Husted et al., 2013; Wesselius et al., 2013) have provided consistent evidence to show significant association of NS-SNPs with altered osteoporosis risk and fracture incidence. More specifically, the NS-SNPs resulting in loss of functional mutations such as G150R, R307Q, E496A, and I568N are associated with reduced bone mineral density, increased bone loss, or high fracture incidence while, by contrast, the NS-SNP mutation leading to gain of function, A348T, is associated with increased bone mineral density and lower fracture incidence. Perhaps, the most informative observations with respect to the role of P2X7R in the osteoporosis disease mechanism have been provided by a recent study comparing the bone properties in mice expressing the P27R carrying P451 or L451 residues (Syberg et al., 2012). The BALB/cJ and 129X 1/2J mice expressing the P451 P2X7R with a large pore-forming ability displayed stronger femurs and higher expression level of C-telopeptide collagen, a bone resorption marker, than C57Bl/6 and DBA/2J mice expressing the L451 P2X7R defective in large pore formation, suggesting that the large pore function is important in regulating the resorption levels and bone remodeling.

In summary, genetic linkage studies and functional characterization of NS-SNP mutations have provided compelling evidence to disclose P2X7R-dependent large pore formation as a previously unrecognized molecular mechanism that underlies the sensitivity to chronic pain and the susceptibility to osteoporosis. Such a mechanism may offer a more specific and promising therapeutic target stratifying treatments of chronic pain and osteoporosis.

## Concluding Remarks

The mammalian P2X7Rs were identified at the molecular level nearly two decades ago. Studies since then have accumulated a large body of evidence to support an important role for the P2X7Rs in mediating extracellular ATP signaling in health and disease. The potential for developing therapeutics targeting the human P2X7R remains high. As discussed in this review, the recent breakthroughs in elucidating the atomic structures of the truncated zebrafish P2X4.1R in the closed and ATP-bound open states have provided the long-awaited structural information that is needed to gain a better understanding of the molecular mechanisms underlying ATP-binding, ion permeation, large pore formation, and activation of the mammalian P2X7Rs. Certainly one should bear in mind that interpretations based on the current crystal structures of the zebrafish P2X4.1R need to exercise cautions as the missing cytosolic N- and C-terminal domains may significantly alter the conformational changes and in particular the movements of the transmembrane domains during receptor activation. No doubt, the structures of a full-length mammalian P2X7R in the closed and ATP-bound open states are required to provide a mechanistic insight into the receptor functions, and particularly the role of the P2X7R specific C-terminal domain (Costa-Junior et al., 2011) and residues in this domain in receptor facilitation and large pore formation (Jiang et al., 2005; Young et al., 2007; Roger et al., 2008, 2010a; Sorge et al., 2012; Xu et al., 2012). The human *P2RX7* gene is highly rich in NS-SNPs, and characterizations of the NS-SNP mutations identified in various disease conditions have revealed additional molecular determinants for the human P2X7R functions and, more importantly, have provided new insights into disease mechanisms. It is anticipated that our increasing understanding of the structure-function relationships of the mammalian P2X7Rs and of the P2X7R-mediated disease mechanisms will facilitate continuing efforts to exploit research on the bench for the benefit of patients at the bedside.

## Conflict of Interest Statement

The authors declare that the research was conducted in the absence of any commercial or financial relationships that could be construed as a potential conflict of interest.
